# Developing sequentially trained robust Punjabi speech recognition system under matched and mismatched conditions

**DOI:** 10.1007/s40747-022-00651-7

**Published:** 2022-06-02

**Authors:** Puneet Bawa, Virender Kadyan, Abinash Tripathy, Thipendra P. Singh

**Affiliations:** 1 Centre of Excellence for Speech and Multimodal Laboratory, Chitkara University Institute of Engineering and Technology, Chitkara University, Punjab, India; 2 Speech and Language Research Centre, School of Computer Science, University of Petroleum and Energy Studies (UPES), Energy Acres, Bidholi, Dehradun, Uttarakhand 248007 India; 3 Department of Computer Science and Engineering, Raghu Engineering College, Visakhapatnam, India

**Keywords:** Sequence discriminative training, Children speech recognition, Data augmentation, Mismatched conditions

## Abstract

Development of a native language robust ASR framework is very challenging as well as an active area of research. Although an urge for investigation of effective front-end as well as back-end approaches are required for tackling environment differences, large training complexity and inter-speaker variability in achieving success of a recognition system. In this paper, four front-end approaches: mel-frequency cepstral coefficients (MFCC), Gammatone frequency cepstral coefficients (GFCC), relative spectral-perceptual linear prediction (RASTA-PLP) and power-normalized cepstral coefficients (PNCC) have been investigated to generate unique and robust feature vectors at different SNR values. Furthermore, to handle the large training data complexity, parameter optimization has been performed with sequence-discriminative training techniques: maximum mutual information (MMI), minimum phone error (MPE), boosted-MMI (bMMI), and state-level minimum Bayes risk (sMBR). It has been demonstrated by selection of an optimal value of parameters using lattice generation, and adjustments of learning rates. In proposed framework, four different systems have been tested by analyzing various feature extraction approaches (with or without speaker normalization through Vocal Tract Length Normalization (VTLN) approach in test set) and classification strategy on with or without artificial extension of train dataset. To compare each system performance, true matched (adult train and test—S1, child train and test—S2) and mismatched (adult train and child test—S3, adult + child train and child test—S4) systems on large adult and very small Punjabi clean speech corpus have been demonstrated. Consequently, gender-based in-domain data augmented is used to moderate acoustic and phonetic variations throughout adult and children’s speech under mismatched conditions. The experiment result shows that an effective framework developed on PNCC + VTLN front-end approach using TDNN-sMBR-based model through parameter optimization technique yields a relative improvement (RI) of 40.18%, 47.51%, and 49.87% in matched, mismatched and gender-based in-domain augmented system under typical clean and noisy conditions, respectively.

## Introduction

In real-life applications, a speech signal heard in one’s ear is a continuous mixture of different kind of signals which are originated from diverse environments and recording conditions. These adverse conditions greatly impact the performance of state-of-the-art recognition systems due to presence of unwanted information in an input speech signal [1]. Despite this, humans are well able to distinguish different sounds from multiple sources. In recent years, communicating with speech-oriented technological devices has become a part of daily usage for billions of people around the globe in form of voice assistant applications: Amazon Alexa, Apple Siri, Google Assistance, Microsoft Cortana [2, 3], etc. Moreover, human generally feel more comfortable by communicating in their native language and thus making use of their devices in real-life applications: military operations, education and medical research. These social interactions help parents in teaching their children in own native language which eventually preserve two objectives: (i) keeping first language alive, (ii) preserving literature and the pride of cultural roots. Nowadays, languages are depleting at an alarming rate, in fact, it has been reported that within next century, almost 50% of existing languages enter into an endangered stage [4]. Although, during spread of Covid-19 pandemic, a demand for deployment of children-based speech technologies have become an important as well as challenging task for various researchers. Many research works have been presented on adult, children, or mismatched training and testing conditions with an objective of removing the constraints of validating speech styles, classes, vocabularies as well as distorted channels [5–8]. These variations in children’s speech arises an urge for developing a robust ASR system which will help in reflecting the future of human–computer interactions in form of technological education. The other major challenge is building of children ASR system in their native language. To fulfill this requirement, one must have effective resource (in form of application or device interface) as well as adequate quantity of training data.

Hence, many approaches for artificial augmentation-based generation of synthetic data under real-life adverse conditions have been worked upon for fulfilling objective of large training data requirement. Likewise, some researchers have investigated automatic recognition of children’s speech under mismatched conditions, i.e., training adaptation to adult speech corpus but it has been a well-known challenging problem due to acoustic differences in speech of adult and child speakers [9–11]. Apart, feature extraction exhibits a compact representation of an input speech signal which is a foremost step in development of an efficient ASR system. Since it is not feasible to recognize speech signals from digitized waveforms due to large-scale variations, thereby, better aspect of the application of noise-robust feature extraction techniques is required to be considered such that the variability among matched or mismatched systems is removed. The extracted feature vectors are though well efficient in capturing relevant information while discarding the redundancies originated due to presence of noise in an input speech signal. Therefore, various feature extraction techniques: RASTA-PLP [12], MFCC [13], GFCC [14] and PNCC [15] have been investigated by various researchers with an effort of deploying an effective noise-robust ASR system. For the past many years, HMM has been a widely adapted modeling technique for efficient learning of parameters corresponding to an acoustic model [16]. With progressive developments in ASR, the flexibility and prediction power of deep learning algorithms have enabled researchers to generate observational probabilities for different HMM states. However, most of the hybrid DNN–HMM architectures being trained for development of speech recognition have been based upon their individual classification of frames or on the basis of cross-entropy. It helped in reduction of frame error rate. Apart, the generation of speech which further processed using method of ASR is considered a sequence classification problem. Therefore, to better match the decision rule in case of matched and mismatched system, various forms of sequence-discriminative training: MMI, MPE, bMMI and sMBR training criteria by employing lattices are being evaluated by earlier researchers [17–20]. The resultant techniques utilized GMM–HMM- and DNN–HMM-based architecture which has resulted into the continuous gains [21]. Subsequently, researchers have expressed some of the disagreements pertaining to comparative analysis of resultant techniques where MMI has been performed better than that of MPE [22] and in [23, 24] sMBR which resulted into effective gains in terms of accuracy. Later, these front- and back-end approaches capabilities are employed in Indian Punjabi language.

In this paper, a robust Speaker-Independent ASR framework has been presented on four front-end approaches: MFCC, RASTA-PLP, GFCC and PNCC to explore effectiveness of Punjabi speech recognition system on various matched (adult speech in train and test) or mismatched (per-mutational mix of adult and child speech in train and test sets) systems. While in Punjabi language, the work on children speech is almost zero and in adult at infant stage because of the non-availability of child and very less adult speech and text-labeled dataset. The implementation has been performed on large adult data and very less children speech on original and synthetic noise injected at lower SNRs. The impact of various types of noises—Volvo, babble, pink, white and factory has been analyzed alone or through pooling of all noise dataset. Likewise, the problem of data scarcity has also been overcome through creation of synthetic noise dataset by pooling it with original corpora using out-data augmentation [25] strategy. Accordingly, in this research, the training child data have been augmented with enough available or self-created adult data with an effort of handling the problem of data scarcity and tried to boost the performance of system in mismatched conditions. Moreover, the geometry of vocal organs of child and adult differs considerably (smaller in children), which arises an urge for scaling of fundamental frequency or pitch. Thereby, an effort for removal of inter-speaker variations and mismatch conditions among test and train datasets has been investigated by utilization of the methodology of VTLN [26]. In addition, the acoustic optimization methodologies based upon the procedure of inter-frame-based discriminative training have been further employed and the performance is monitored for each framework on hybrid front-end techniques.

The remainder part of the paper is organized as follows: the next section describes the related work, and in the third section, the technologies study employed are discussed. The fourth section presents corpus use, and in the fifth section, proposed approaches on heterogeneous robust ASR framework using sequencing discriminative training are processed in matched and mismatched systems using original and synthetic dataset are outlined. The sixth and the last sections present experimental study along with conclusion and future work, respectively.

## Related work

Earlier adaptations for development of an ASR system were based upon the interpretation of phonemes for effective creation as well as recognition of vowel sounds. However, development of noise-robust ASR systems has been greatly affected by acoustic environments in the presence of background noise, reverberation and other distortions caused due to interfering signals [27]. The primary requirement of increasing efficiency of an ASR system in native language is important and, basically, dependent upon the representation of compact information by utilizing various technique of filtering noise and undesired information present in an input speech signal. Gong et al. [28] analyzed the impact of noisy conditions on building a robust ASR system by portraying a survey of 250 publications related to the techniques while discarding undesired information present in form of noise from an input speech signal. The researcher highlighted the importance of categorization based on measurement and analysis of noise-resistant features. The techniques for speech enhancement and hidden Markov model adaptation for the compensation of unwanted noise are also presented in it. Likewise, Diethorn et al. [29] highlighted the use of noise-reducing processors in modern daily-life communication systems. It consists of telephone handsets, mobile phones, teleconferences and in-home-based telephonic appliances and speaker phones. The researcher focused on the methods of extracting useful information from a single-channel noisy system by utilizing the techniques of short-time spectral modification. Further, Farahani et al. [30] based upon the higher value of SNR highlighted the minor adaptations in a signal with performance degradation due to mismatch conditions between train and test set. The researcher highlighted the replacement of speech signal features with features generated on the basis of autocorrelation sequence. Ma et al. [31] experimented on novel noise reduction algorithm by utilization of Wiener gain function by exploring it on bias and variance properties of the multi-taper spectrum. MFCC has been a broadly used method for feature extraction; however, degradation of performance of most of the system is seen under noisy conditions. Kadyan et al. [32] presented heterogeneous feature vectors using MFCC and PLP fusion along with RASTA which further utilized GA + HMM- and DE + HMM-based hybrid classifiers under both clean and real conditions. The researchers concluded with an overall improvement using hybrid classifiers by ~ 13% with MFCC and DE + HMM when compared with RASTA-PLP. On the other hand, many advanced noise-robust features: GFCC, PNCC and their comparative analysis with different SNRs have been presented by Zhang et al. [33]. The outcome revealed noise robustness and effectiveness of PNCC feature extraction methodology under lower SNRs as compared to that of traditional modifications of feature vectors using MFCC and GFCC approaches. Moreover, the concatenated features GF-MFCC for performance improvement in both clean and noisy environments were investigated by Dua et al. [34]. Since, the speech recognition was named as a sequence classification problem such that there is an effective need for consideration of inter-frame constraints that helped in optimization of HMM parameters alongside the phonetic word references and powerful language model. On the other hand, the estimation of HMM model parameters was made by maximizing the likelihood when the states of model were paired in a supervised manner [35]. However, Nádas et al. [36] concluded that non-consideration of other possible word strings during MLE training frequently leads to an increase in likelihood of word corresponding to its transcribed utterances. Later Povey et al. [37] experimented the comparison of the use of another discriminative training MMIE and generally utilized MLE. The outcomes showcased a significant increase in the performance of the system using MMIE as compared to that of MLE on very large data sets. Finally, Veselý et al. [22] represented the comparison of different sequence-discriminative training criteria: MMIE, MPE, sMBR and bMMI. The outcomes of the comparison have demonstrated an average relative improvement of 8–9% by utilizing the cross-entropy-based DNN model. Finally, in this paper, an effort has been made to analyze the characteristics of different front and back-end approaches on less resource language like Punjabi. Consequently, processing of inter-speaker variability in test set along with efficient modeling of model parameters with various discriminative approaches on train set.

## Theoretical background

### Feature extraction

**Fig. 1:**
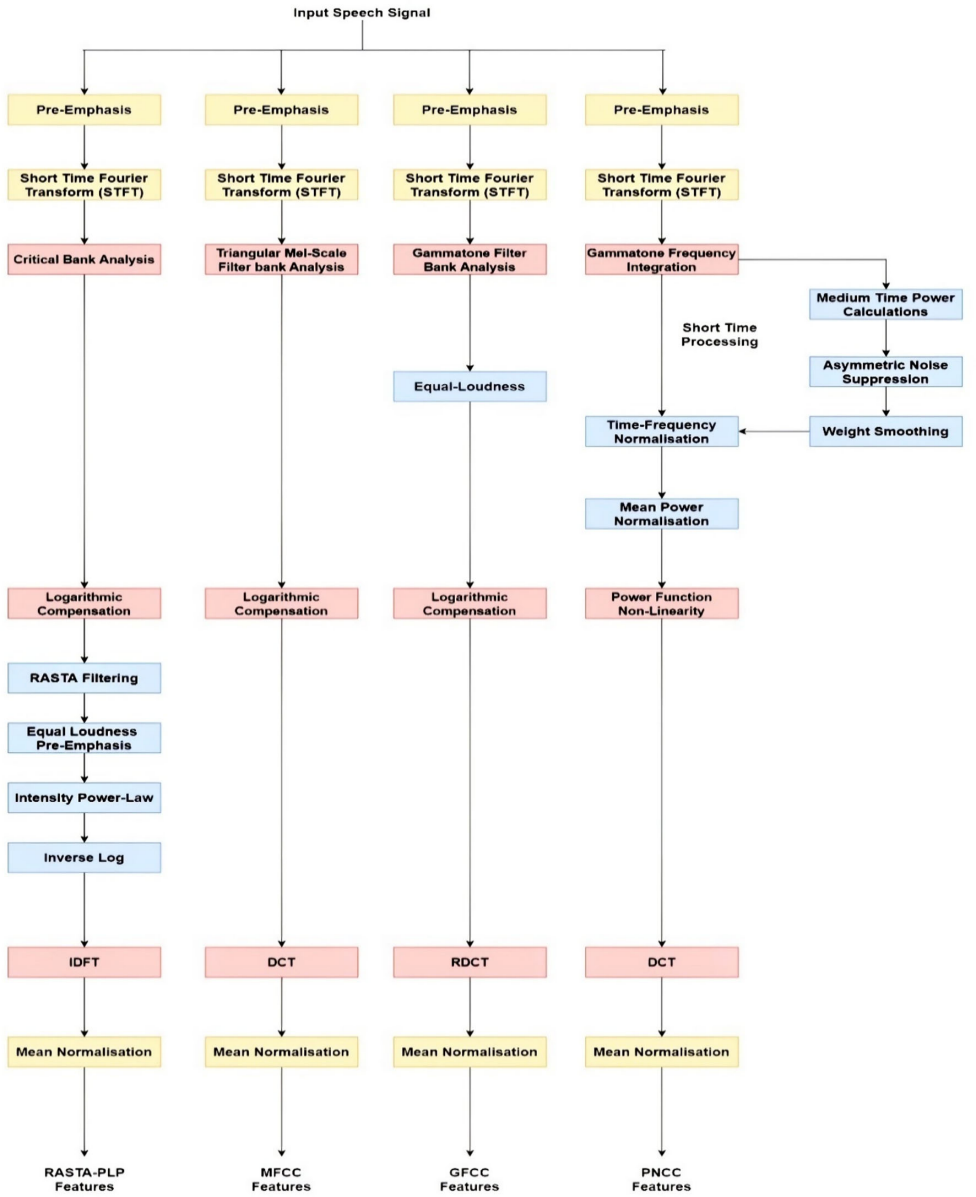
A comparative block diagram of heterogeneous front-end feature extraction approaches

The production of feature vectors is one of the mandatory task that helps in the development of a noise-robust ASR system while preserving relevant information of an input speech signal. Thereby, various robust techniques: MFCC, RASTA-PLP, GFCC and PNCC have been adopted to represent the spectral envelope of a model as shown in Fig. 1.

It is having different capacity of handling required information while discarding unwanted information present in an input speech signal. MFCC has been one of the widely used dominant methods for frame-by-frame extraction of spectral features. The specific parameters for calculation of speech spectrum are related to a frame sequence of *N* frames in an input signal $s(n,t)$ over time period *t* which are acquired by the use of Fast Fourier Transform (FFT) using the following equation:

1 $$C\left(n,t\right)=\sum_{n=0}^{N-1}\mathrm{log}\left(\sum _{n=0}^{N-1}s\left(n,t\right)\times {e}^{-2\pi jkn/N}\right){e}^{-2\pi jkn/N}.$$

In RASTA-PLP, the spectral amplitude is changed using methodology of the compression of non-linear transformation followed by computation of the critical-band power spectrum as performed in PLP. This assesses into smoothing of momentary noise variations which are present in signals.

It has utilized 17 band-pass IIR filter-channel [12] and corresponding parameters are computed using all-pole model as shown in the following equation followed by the procedure of inverse logarithm to acquire relative spectrum:

2 $$H\left(z,t\right)=\left(0.1\right)\times \frac{2+{z}^{-1}-{z}^{-3}-2{z}^{-4}}{{z}^{-4}\times \left(1-0.98{z}^{-1}\right)}.$$

In GFCC, the windowed signal is processed through a 64-Gammatone channel filter bank corresponding evaluation of the central frequency ${f}_{\mathrm{center}}$ is given by time *t*, for which the impulse response is computed through the following equation:

3 $$g\left(f,t\right)=a{e}^{-\frac{2\pi }{{tb}_{\mathrm{cm}}}}\times \mathrm{cos}\left(2\pi {f}_{\mathrm{center}} + \varphi \right).$$

Further, the evaluation of derived filter is represented which are equivalent to rectangular bandwidth (ERB) through the following equation. Finally, separation of an ambient noise in an input signal is performed by taking the cube root of time–frequency (*T*–*F*) representations:

4 $${b}_{\mathrm{m}} = b\times \mathrm{ERB}\left({f}_{\mathrm{center}}\right)= 24.7\left(\frac{4.37{f}_{\mathrm{center}}}{100} + 1\right),$$

whereas the processing of PNCC [15] has made a large impact for consideration of progressively powerful features with respect to acoustical variability and close to human auditory processing. The environmental temporal integration analysis for speech enhancement is performed by estimation of the medium-time power through calculation of the running average of power assessed. It is related to a single frame and is given by

5 $$\underline{Q}\left[m,l\right]= \frac{1}{2M+1}\sum_{{m}^{{\prime}}=m-M}^{m+M}P\left[{m}^{{\prime}},l\right].$$

The negative output for linear high-pass filter in power domain is performed in RASTA-PLP which is basically a significant problem. It results in negative power coefficients. Therefore, the process which makes the use of complete asymmetric non-linear suppression processing along with temporal masking as in the following equation helped in noise suppression:

6 $${\underline{Q}}_{\mathrm{t}}\left[m,l\right]=\left\{\begin{array}{l}{\underline{Q}}_{0}\left[m,l\right], \qquad \quad {\underline{Q}}_{0}\left[m,l\right] \ge {\lambda }_{\mathrm{t}}{\underline{Q}}_{\mathrm{p}}[m-1,l]\\ {\mu }_{\mathrm{t}}{\underline{Q}}_{\mathrm{p}}\left[m-1,l\right], \quad {\underline{Q}}_{0}\left[m,l\right]< {\lambda }_{\mathrm{t}}{\underline{Q}}_{\mathrm{p}}[m-1,l]\end{array}.\right.$$

Moreover, the impact of ANS and temporal masking are as shown in the following equation for a given time and frequency which can be represented using process of smoothing of weights:

7 $${\underline{R}}_{\mathrm{p}}\left[m,l\right]=\left\{\begin{array}{l}{\underline{Q}}_{0}\left[m,l\right], \qquad \quad {\underline{Q}}_{0}\left[m,l\right]\ge {\lambda }_{\mathrm{t}}{\underline{Q}}_{\mathrm{p}}[m-1,l]\\ {\mu }_{\mathrm{t}}{\underline{Q}}_{\mathrm{p}}\left[m-1,l\right], \quad {\underline{Q}}_{0}\left[m,l\right]<{\lambda }_{\mathrm{t}}{\underline{Q}}_{\mathrm{p}}[m-1,l]\end{array}. \right.$$

This process is evaluated using weighted average function which is computed over an average relation of transfer function corresponding to its ANS and temporal integration using the equation as follows:

8 $$S\left[m,l\right]=\left(\frac{1}{{l}_{2}-{l}_{1}+1}\sum _{\mathrm{l}={l}_{1}}^{{l}_{2}}\frac{\underline{R}\left[m,{l}{^{\prime}}\right]}{\underline{Q}\left[m,{l}^{{\prime}}\right]}\right).$$

### Acoustic modeling and parameter optimization approaches

The estimation of posterior probability in corresponding to HMM states is basically performed by the process of training of DNN–HMM system. HMM is a widely used model that works in such a way that the transitional probabilities between all the possible states of the model are contained by a Markov Chain. In general, DNN is a feed-forward neural network composed of a large number of hidden layers which are subsided between its input and output layers. Therefore, a logistic function mapped to a layer below *x* for corresponding utterance u at time t in particular HMM states can be represented as

9 $${y}_{u}\left(s,t\right)= \mathrm{logistic}\left(x\left(s,t\right)\right) =\frac{1}{1+{e }^{-{x}_{u} (s,t))}}.$$

Consequently, the class probability ${P}_{u}(s,t)$ for particular utterance $u$ at a given time $t$ of such structure is obtained using a SoftMax nonlinearity using the following equation:

10 $${P}_{u}\left(s,t\right)=\frac{\mathrm{exp}\left\{{a}_{u}\left(s,t\right)\right\}}{\left(\sum_{{s}^{{\prime}}}\mathrm{exp}\left\{{a}_{u}\left(s,t\right)\right\}^{\prime}\right)},$$

where ${a}_{u}(s,t)$ corresponds to an activation function corresponding to output layer at a particular HMM state $(s)$. Therefore, an optimization of a given objective function is usually trained using a standard error-back propagation procedure [13]. It is performed by evaluating a natural cost function C as demonstrated in the following equation by utilizing SoftMax output function. It tried to employ a cross-entropy between target probability ${d}_{u}(s,t)$ (generally, value is zero or one) and probabilistic output of SoftMax nonlinearity as evaluated in Eq. (10):

11 $$C =-\sum _{u=1}^{U} \sum _{t}{d}_{u}\left(s,t\right)\mathrm{log}\left({P}_{u}\left(s,t\right)\right).$$

#### Maximum likelihood estimation (MLE)

The most common methodology of maximum likelihood estimation (MLE) is generally utilized to learn the parameters $\theta$ corresponding to HMM with an objective function given by

12 $${F}_{\mathrm{MLE}}\left(\theta \right)=\sum_{u=1..U}\mathrm{log}{P}_{\theta }\left({X}_{u}|M\left(u\right)\right),$$

where $u$ is total number of utterances corresponding to its training set and ${X}_{u}$ is an observation for $M(u)$ graph of all possible words and sequences in transcription ${X}_{u}$.

#### Maximum mutual information estimation (MMIE)

The method of MLE somewhere prompts the over-estimation in assessed transitional probabilities. Along these lines, a methodology of maximum mutual information estimation (MMIE) is utilized with a scaling fudge factor $\kappa$ to make up for the over-estimation from the frame wise likelihood. Subsequently, the likelihood identified with reference transcription is adjusted which further tried to utilize MMIE function that is being modeled as

13 $$\begin{aligned}{F}_{\mathrm{MMIE}}\left(\theta \right)& =\sum_{u=1}^{U}\mathrm{log}{{P}_{\theta }\left({X}_{u}|M\left(u\right)\right)}^{k} P(M|u) /\\ & \quad \sum_{{w}{^{\prime}}}{P}_{\theta }{\left({X}_{u}|M\left(w^{\prime}\right)\right)}^{k} P(w^{\prime})).\end{aligned}$$

On the other hand, it is well known that the objective function for MMI estimation is a sequence-based discriminative training where the posterior probability of a word sequence for a given acoustic is maximized. It is similar as that in forward–backward MLE estimation which is represented using Eq. (12). Likewise, for discriminative aspects, the optimization of the objective function of MMIE is achieved through the process of maximizing the numerator along with increasing the likelihood of correct word sequence. In addition, the denominator is minimized by decreasing the total likelihood of all other word sequences unlikely.

#### Minimum phone error/minimum word error

It is well known that the MMIE estimation in Eq. (13) is sentence-level, thereby the basic idea behind MPE/MWE has a direct relation to the sub-sentence, i.e., (words or phones):

14 $$\begin{aligned}{F}_{\frac{\mathrm{MPE}}{\mathrm{MWE}}}\left(\theta \right)&=\sum_{u=1}^{U}\mathrm{log}{{P}_{\theta }\left({X}_{u}|M\left(u\right)\right)}^{k} P({M}_{w}|u)A(w,{w }_{u}))\\ &\quad \times \sum_{{w}{^{\prime}}}{P}_{\theta }({X}_{u}|M(w^{\prime}))P(w^{\prime}) ),\end{aligned}$$

where *A*(*w*,*w*_*u*_) corresponds to the phone/word transcription accuracy of a sentence w for a given reference *w*_*u*_. Therefore, the optimization function for MPE/MWE with the context of given sentence reference is made by evaluation of the probable sentences with lower phone error rates.

#### State-level minimum Bayes risk (sMBR)

Minimizing the error rate, which is calculated corresponding to the HMM state topology along with its language model, is performed by utilizing the procedure of state-MBR (sMBR). Thereby, the model represented is similar to that of objective function *F*_MPE/MWE_($\theta$) using Eq. (14) where *Q*(*w*,*w*_*u*_) corresponds to the correct state labels accuracy given by

15 $$\begin{aligned} {F}_{\mathrm{sMBR}}\left(\theta \right)& =\sum_{u=1}^{U}\mathrm{log}{{P}_{\theta }\left(M\left(u\right)\right)}^{k}P({M}_{w}|u)Q(w,{w }_{u}))/\\ &\quad \sum_{{w}{^{\prime}}}{P}_{\theta }({X}_{u}|M(w^{\prime}))P(w^{\prime})).\end{aligned}$$

#### Boosted maximum mutual information estimation (bMMIE)

The optimization function of boosted-MMI estimation is a modification of the MMIE function as in Eq. (13) with an objective of boosting the likelihood of the path with more error and is represented as in the following equation:

16 $$\begin{aligned} {F}_{\mathrm{bMMIE}}\left(\theta \right)&=\sum_{u=1}^{U}\mathrm{log}{{P}_{\theta }({X}_{u}|M\left(u\right))}^{k} P\left(u\right)\\ &\quad \sum_{w{^{\prime}}}{P}_{\theta }{\left(M\left(w^{\prime}\right)\right)}^{k}P\left(w^{\prime}\right){e}^{-bA({w}{^{\prime}}, u)}), \end{aligned}$$

where *b* corresponds to the boosting factor and can be evaluated at the word/phone level whereas the formulation of the boosting likelihood paths at the state level can be evaluated as in the following equation:

17 $$\begin{aligned}{F}_{\mathrm{bMMIE}}\left(\theta \right)&=\sum_{u=1}^{U}\mathrm{log}{{P}_{\theta }({X}_{u}|M\left(u\right))}^{k} P\left(u\right)\\ &\quad \sum_{w{^{\prime}}}{P}_{\theta }{\left(M\left(w^{\prime}\right)\right)}^{k}P\left(w^{\prime}\right){e}^{-bQ({w}{^{\prime}}, u)}). \end{aligned}$$

## Experimental overview

### Dataset details

The experiments have been performed on different Punjabi corpus which are composed of both adult and children corpora. The adults’ ages range from 17 to 30 years with 22 speakers which have been recorded using a microphone in a clean environment. Though adults are more trained than children, it is somewhat difficult to collect the efficient data required for developing robust children ASR systems. Therefore, a smaller number of 39 children’s speakers which range from 7 to 12 years have been recorded taken with and without the use of a microphone. All the recordings for both adult and children speech signals are sampled at 16 kHz utilizing 40 non-silence phones and further the utterances are transcribed in reference to the speaker-wise segmented dataset using an open-source software package, i.e., Praat [38]. Likewise, for handling the silence existing in the corpora, the use of silence phones file along with oov.txt in Kaldi by including the silent word, being termed as “ < !SIL > ” for the back-end process of efficient resource management configuration has been done. Thus, an arbitrary word from vocabulary has been chosen and likewise the selected silence word further has no influence or impact on existing vocabulary set of phonemes/lexicons being employed for training the system The summary constituting the more information on datasets is detailed in Table 1.

**Table 1:** Detailed information of Punjabi adult and children corpora

Characteristics	Adult dataset	Child dataset
No. of speakers	21	39
Speech data type	Isolated words and phonetically rich sentences	Continuous speech sentences
Recording environment	Closed room using dictaphone and microphone	Open and closed environment using microphone
No. of utterances	3953	2159
Age	17–26 years	7–12 years
Duration	10 h 12 min	4 h 10 min
No. of unique words	6567	4863
Gender	9 male/12 female	20 male/19 female

Further *n*-gram language modeling is trained which depends upon the last $(n-1)$ words as in the following equation. This helped in evaluating the likelihood of the word sequence corresponding to a particular utterance through a transcribed dataset.

18 $$P({w}_{1},{w}_{2},{w}_{3}\dots {w}_{L})\approx \prod_{j=1}^{L}P({w}_{j}| {w}_{j-2},{w}_{j-1}).$$

Currently, large number speech data as well as the resources have been experimented on adult dataset. On the other hand, nearly zero effort has been made for developing children’s speech recognition systems in native languages like Punjabi. Therefore, the work has been divided depending upon the use of four systems as shown in Table 2.

**Table 2:** Different matched or mismatched system employed for training and testing

Type of ASR	Training	Testing
Adult ASR-S1 system	Adult dataset	Adult dataset
Children ASR-S2 system	Children dataset	Children dataset
Mismatched ASR-S3 system	Adult dataset	Children dataset
Semi-mismatched-S4 system	Adult and children mixed dataset	Children dataset

### Noise augmentation

**Fig. 2:**
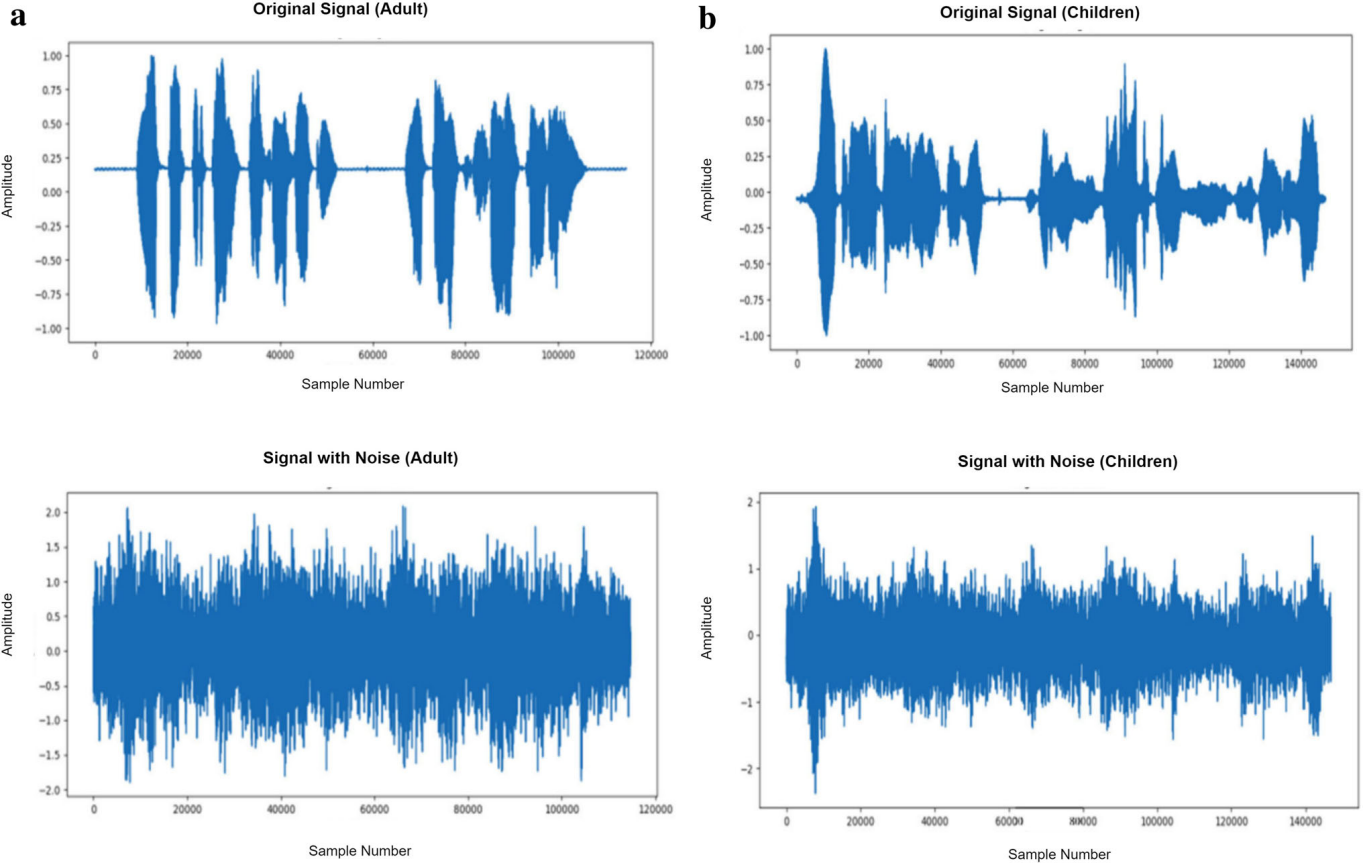
**a** Adult original signal and noisy signal. **b** Child dataset original signal and noisy signal

The essentiality is in learning the disentangled representations of an audio signal in the presence of the background noise being injected at lower SNR. Thus, four different variations of the noise—factory, babble, white and pink noise are taken from standard NOISEX-92 database [39]. These noises have been injected at different SNR values in Eq. (19) into the original clean dataset as detailed in Table 1. The sox command through python [40] has been used to inject the noise file into the input clean audio file. It generates noise augmented samples by matching the sampling rate and duration of an input file of an adult dataset as shown in Fig. 2a and child dataset as shown in Fig. 2b with respect to its original clean speech signal.

19 $${\mathrm{SNR}}_{\mathrm{dB}}= 10 \times \mathrm{log}10 \frac{\mathrm{Ps}}{\mathrm{Pn}}.$$

### Spectral augmentation

**Fig. 3:**
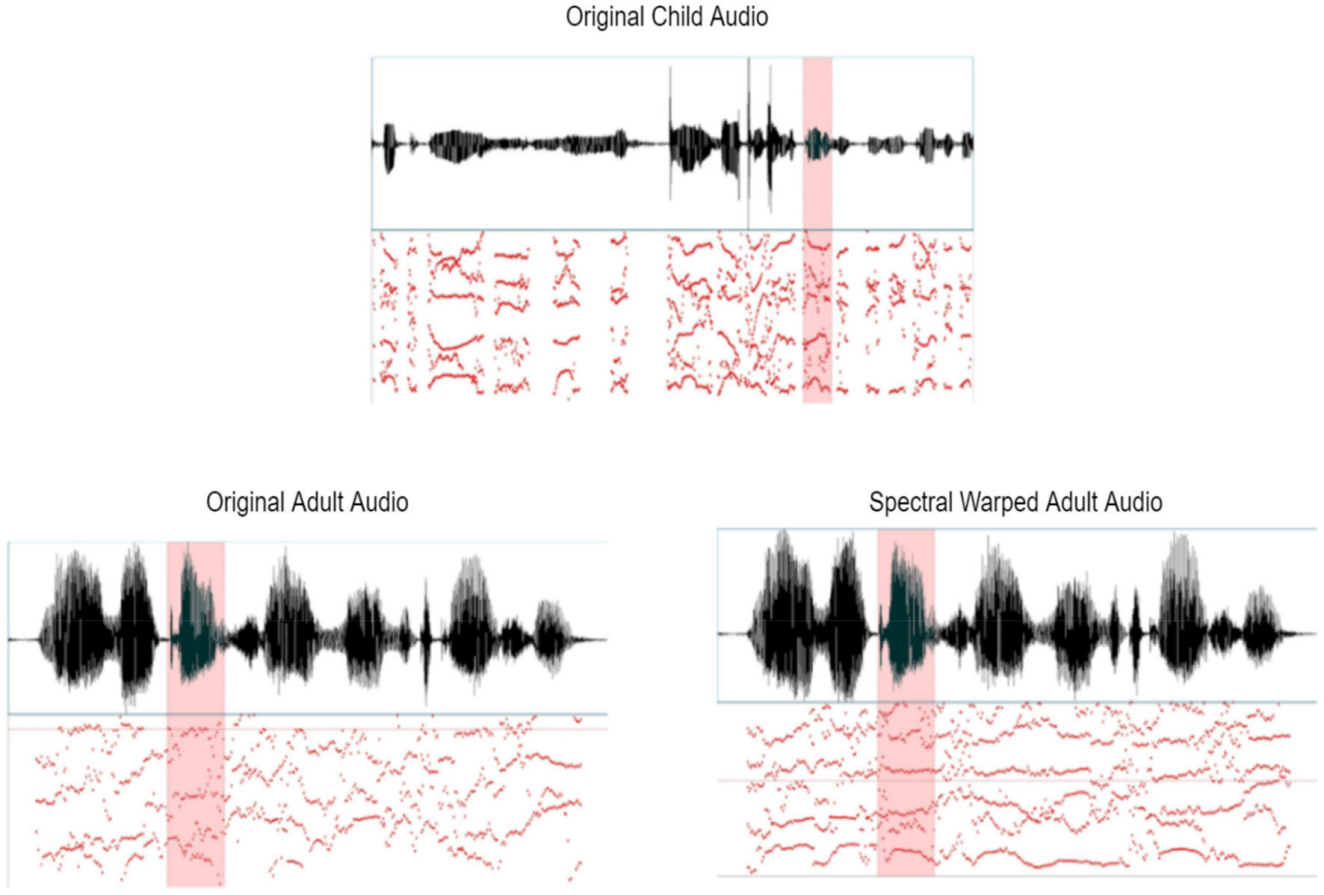
Comparative illustration of original children and spectral warped adult audio signal

Conceptually, the warping factor is the ratio between the length of a speaker’s vocal tract and some idea of a reference vocal tract length. However, for calculating the length of a speaker’s vocal tract from acoustic data is always a challenging task. Therefore, a certain warping factor ranging from the values of − 0.20 to 0.20 with the step size of + 0.05 has been chosen in this study with an aim of maximizing the probability corresponding to the normalized features. Under noisy conditions it provides a particular notion for use of adequate statistical model. Figure 3 illustrates the reference-derived spectral warped audio for an adult is compared to children audio such that an assumption for variation among the lip movements under the mismatched condition shows that it do not affect the estimated warping factor.

## System overview

The processing of clean speech always generates better output in any ASR system but it becomes challenging with real environment or synthetic noisy dataset. The real-life speech when tested on a clean train system degrades the performance of the ASR system. Therefore, an effort has been made to evaluate the characteristics of different front-end approaches to find an optimal approach that can yield better output for both types of system (generally the system with environment differences between train and test set) using Kaldi toolkit [41]. Initially, the original clean signal is injected with different types of artificial noise using Algorithm 1. It is possible through augmentation strategy which tries to fulfill the requirement of data scarcity problem of training dataset. Later pooling of such dataset has been performed such that Fig. 1 demonstrates the method of noise-based data augmentation through injection of background noise at varying SNRs into different combinations of clean datasets as detailed in Table 1. Although the perception of an individual identifying with respect to frequency context present in corresponding signal is elucidated to be non-linear. The case of machine processing a real-world input speech signal is always challenging due to various inbuilt parameters like environment, speaker and other acoustic features. To tackle such issue at training and test end, initially four front-end feature extraction approaches: MFCC, RASTA-PLP, GFCC, and PNCC are being investigated with a target of extracting robust feature vectors that helped in extraction of relevant information in spite of the presence of noisy background. The main focus of the feature extraction process is the improvement of cepstral representation by extraction of information which is nearly close to the human perception. First, conventional MFCC is a widely used feature extraction approach which is based upon the typical 40-channel Mel-Filterbank with a frame size of 25 ms and frame shift of 10 ms. The perceptual sensitivity on the magnitude axis is taken into account by expressing magnitude upon log-spectrum motivated by the use of mel-scale. However, MFCCs are not robust to noise such that the performance is degraded in the presence of an additive noisy environment. Second, RASTA-PLP is more robust to steady-state spectral features. In this technique, the temporal derivatives of critical log-spectrum are estimated using a regression line based on first-order IIR filtering. Here, while performing the process of integration, the pole of the system ($z=0.98$) through Eq. (2) is initialized. Therefore, the separate channel estimation phase in the process of RASTA-PLP helped in reduction of convolution noise which is quite different from that of processes being involved in techniques with the change in transfer function. However, the accuracy evaluated on the frequency scale is quantized based upon the selection of different criteria of information extraction process. In this way on third, the equivalent of MFCCs, which is GFCC is computed based upon its 64-channel Gammatone filter-bank using a frame size of 25 ms and a frame shift of 10 ms. Later, PNCC is employed which makes the use of typical frame sizes of 25 ms and 10 ms just like MFCC, RASTA-PLP and GFCC approaches. In this process, every frame in a particular audio signal is processed using Povey window [41] and furthermore FFT is being evaluated on 256-bit resolution. The initial processing stages for evaluation of PNCC are quite similar to that of the stages of MFCC and PLP. However, the difference lies in the process of the analysis of frequency performed which are utilized using gamma-tone filters. Further, the long duration temporal analysis accomplished using noise reduction is evaluated by a series of non-linear time which lies on the varied operations being performed.
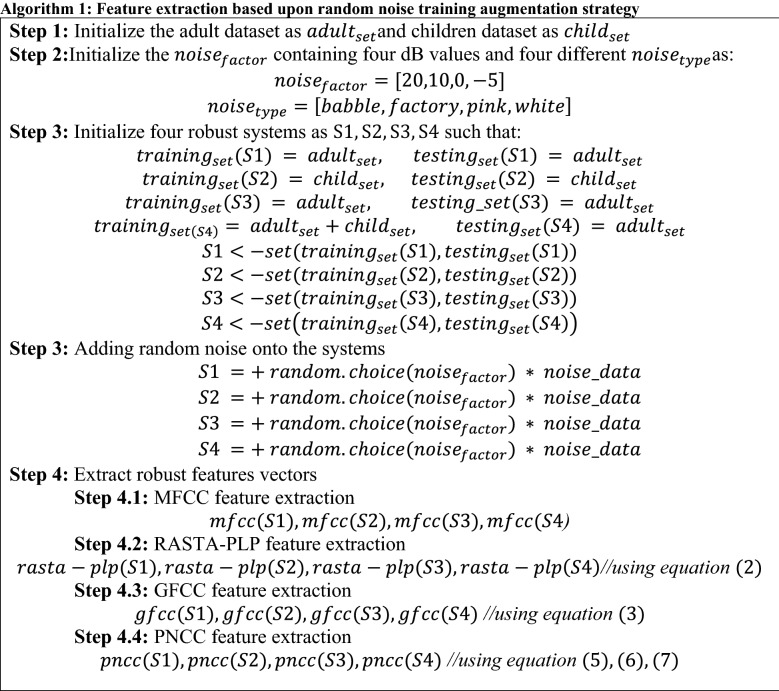


Further, the final refined feature vectors are classified by computing the cepstral mean and variance normalization (CMVN) which are being evaluated using the following equation for each process. It helps in fixing the data samples such that they remain in an appropriate format as required for the process of acoustic modeling.

20 $$c^{\prime}\left(i,t\right)=\frac{\left(s\left(i,t\right)-\mu \left(i,t\right)\right)}{\sigma \left(i,t\right)}.$$

**Fig. 4:**
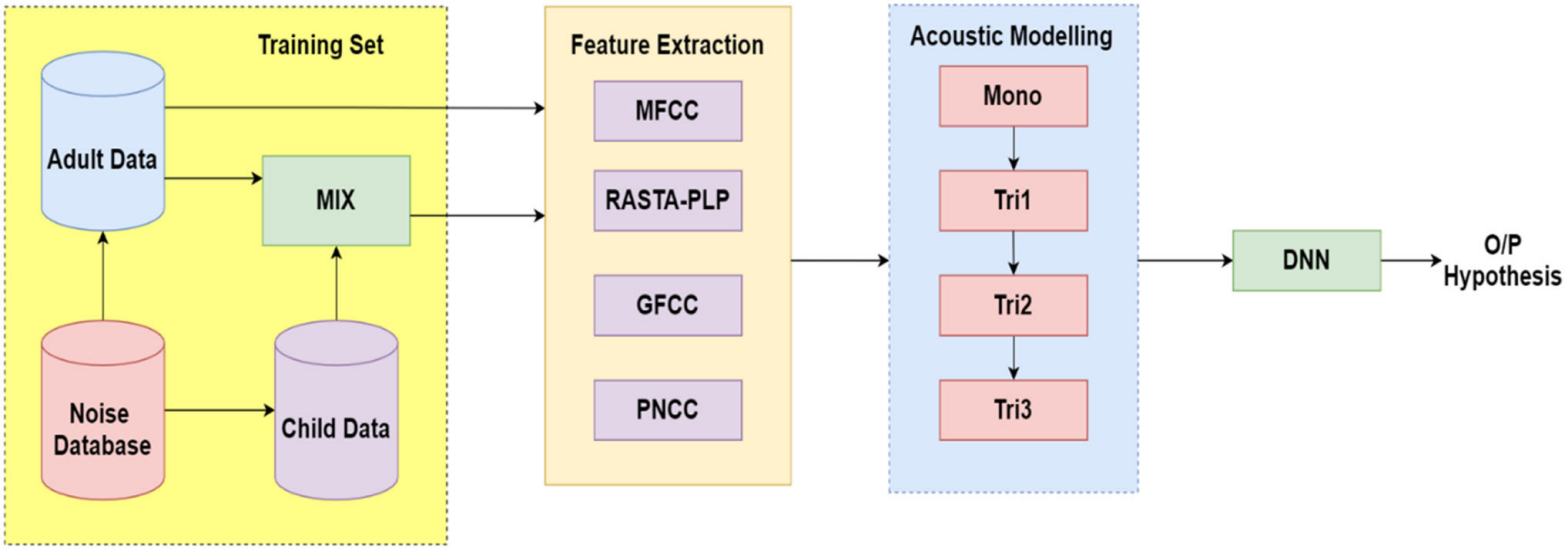
Basic block diagram of heterogeneous feature extraction-based ASR framework on true matched and mismatched systems

These features are further processed to remove inter-speaker variability factors. In the first phase of training procedure, mono-phone (mono) models are generated for very small quantities of data. Further triphone models are trained which includes the process of computation of the delta features (tri1) and delta–delta features (tri2). However, the process of splicing helped in extraction of 13-dimensional features across ± 4 frames. It resulted in generation of 117 dimensional vectors. Thus, it is difficult to evaluate upon a large number of vectors so the procedure of LDA + MLLT (tri3) estimation is applied with an objective to reduce the dimensions from 117 to 30. Finally, a global fMMLR is applied to align the reduced dimensions to normalize the inter-speaker variations. Finally, the different systems are trained on hybrid DNN–HMM acoustic models as shown in Fig. 4.

**Fig. 5:**
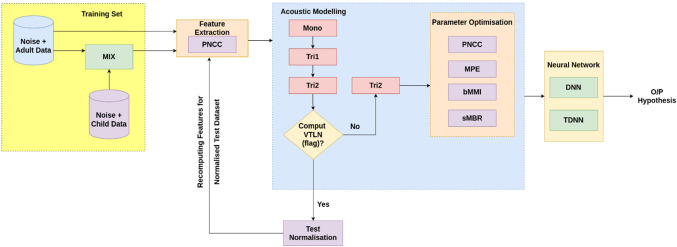
Basic block diagram of robust ASR framework on vocal length normalized-induced front-end approach using varying discriminative sequence training on mismatched systems

**Fig. 6:**
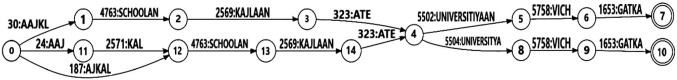
Lattice network for word lattice in the speech utterance

Figure 5 demonstrates the proposed system utilized the noise-robust PNCC features being experimented on a noise-augmented pooled dataset for adults and the combination of adult and child dataset. These extracted features are further normalized using CMVN and further trained on mono-phone (mono) and tri-phones (tri1, tri2, tri3) models as in the baseline system. The inter-speaker variations among children and adults are key parameters which try to enhance the performance of the system. Therefore, the intuitive method of VTLN has been implemented by warping the spectrum in a frequency axis particularly on the test dataset. This type of normalization helps in the reduction of inter-speaker variability by relatively placing the format positions in its normalized spectrum. However, the current ASR systems are mostly trained with MLE and further methods of sequence-discriminative training have been experimented. Moreover, the process of generation of lattices is also employed at the modeling phase which serves as an important aspect. It acts as an intermediate format between interoperation format and the corresponding recognition passes. Lattices related to the certain utterances are created through utilization of the arrangement of back pointers for which a solitary Viterbi back pointer is being stored at the word level. The visualization of the lattice for a Punjabi word sequences is shown in Fig. 6. It is converted into non-compact structural form such that the comparing arcs are being removed alongside the addition of acoustic and language model costs.

While implementing MMI as an objective function for parameter optimization, maximization of the numerator (reference labels) and minimization of the denominator (chance of others) is performed. The generated lattices are expanded to HMM such that different pronunciations for the words are accounted for by just considering only certain word sequences available in the transcript. Finally, the state occupancy probability (*γ*) for both the numerator and the denominator lattice occurrence is separately computed through the following equations:

21 $${\gamma }^{\mathrm{num}}(j,t) = P({q}_{t}=j | {X}_{u},{M}^{\mathrm{num}}),$$

22 $${\gamma }^{\mathrm{den}}(j,t) = P({q}_{t}=j | {X}_{u},{M}^{\mathrm{den}}).$$

Here, the number of iterations (*i* = 1 to 8) for model-space training in MMIE is experimented which helped in the reduction of the likelihood of word sequences apart from the reference utterances. MMI usually works by considering large segments of multiple patterns corresponding to the utterance whereas MPE is focused on the optimization at the sub-`string pattern level. Therefore, the major impact is of phones being implied such that the different language models substituting the value (*n* = 1, 2, 3, 4) in Eq. (18) are being experimented. Moreover, the parameters for boosting the likelihood of the word sequences (boost factor) which range from 0.05 to 0.25 is considered for the process of bMMI. It is well known that the MBR family use for optimization was designed with an objective of minimizing the error rate in reference to the different granularity of labels. The average accuracy of the given states referring to every path of lattice corresponding to reference is evaluated. This helps in the calculation of MBR posterior which is computed over the denominator lattices for utterance through Algorithm 2.
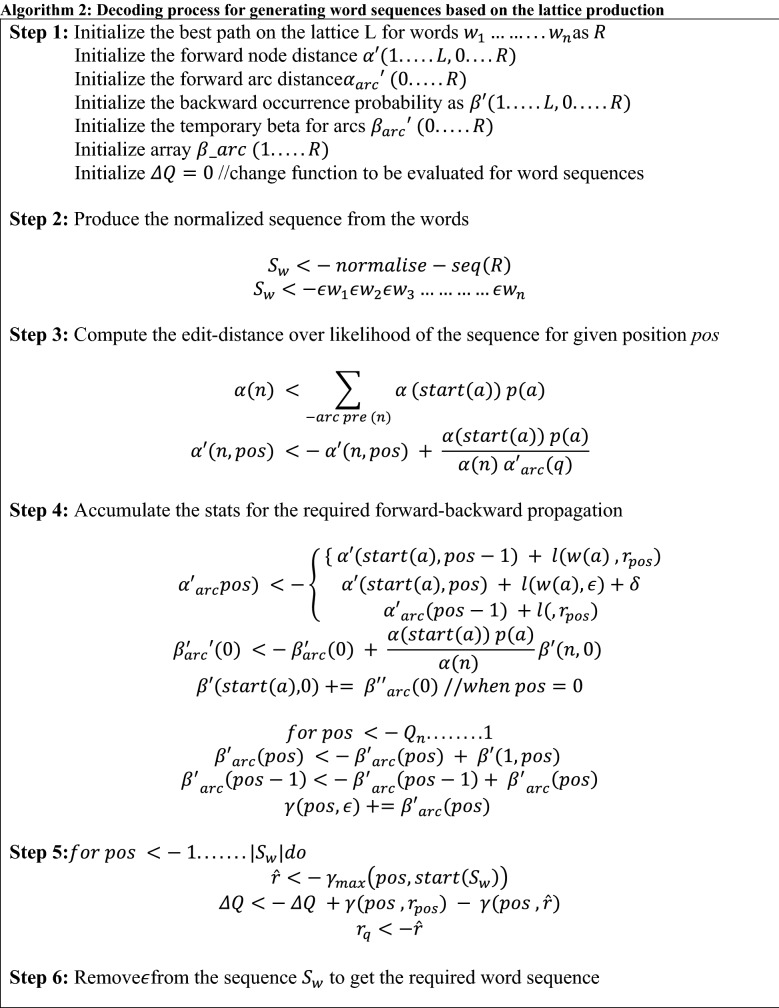


In view of a certain state-etiket, the undermined Markov speaker model requires some very simple expressions: it is simply the product of the probabilities of sound characteristics for every frame which fits into this label (i.e., the acoustic classifier results, known as the acoustic score) and the probability of each frame being multiform. In addition, there may be a much greater state than the label, for example the number of steps taken inside that phone, the mode of the phrase and the preceding words. The significance of using word sequence-based decoding process is being utilized in sequences of separate decision problems involving tiny sets of confusing words for segment lattices being created for developing a general-purpose automated speech recognition (ASR) system. Likewise, in successive rescoring SMBR passes, acoustic models which distinguish between competing words in such classes are subsequently employed. Hence, the refinement of the search area which permits specialized models of discrimination is proven to be an advantage over rescoring with classically trained models of discrimination. Finally, these specialized models of discriminative training involving adult dataset and adult–child mix training are trained on hybrid DNN–HMM models considering both clean and noisy environments as a test set. The key impetus, however, is to train the model, which is effective in capturing long-term dependence between the missing acoustic characteristics. Thus, the capture of these long-term maladjusted relationships became efficient later on with another modified neural network design of TDNN. Thus, both DNN and TDNN architecture are ultimately trained for speed disturbed data via sequence-based training optimization of acoustic modeling. The corresponding performance is represented in the form of Word Error Rate (WER) and RI using the following equations, respectively:

23 $$\mathrm{WER}\left(\%\right)=\frac{S+I+D}{N},$$

24 $$\mathrm{RI}\left(\%\right)= {(N}_{\mathrm{E}}-{O}_{\mathrm{E}})/{O}_{\mathrm{E}}.$$

## Experimental results

### Performance analysis on adult, children and mismatch ASR system under clean environmental conditions

The four-baseline system (S1–S4) has been initially framed by evaluating following systems: true matched (adult train and adult test—S1 system, child train and child test—S2 system), true mismatched (adult train and child test—S3 system) or semi-matched (adult + child train and child test—S4 system). All the systems have been evaluated in clean train and test conditions using conventional MFCC front-end approach only. It has been analyzed from Table 3 that system S1 and S2 performed well on the DNN–HMM acoustic model with a WER of 6.52% and 15.86%. It also showed that the S3 system has large decay of performance accuracy due to acoustic variability among child and adult speech. It represents a WER of 41.28% which is highest among all the systems. In addition, to further enhance the accuracy of the S3 system, a small corpus of children speech has been included in S4 training set and tested on the same test set of S3. It obtained a performance improvement with a R.I. of 10.02% in comparison to that of S3 system.

**Table 3:** WER obtained on different system type using conventional front-end (MFCC) and acoustic model method in clean environment conditions

Training set	Testing set	System type	DNN (WER%) (%)
Adult	Adult	S1	6.52
Child	Child	S2	15.43
Adult	Child	S3	41.28
Adult–child	Child	S4	14.27

### Performance analysis for matched and mismatched ASR system under varying noisy test conditions

**Fig. 7:**
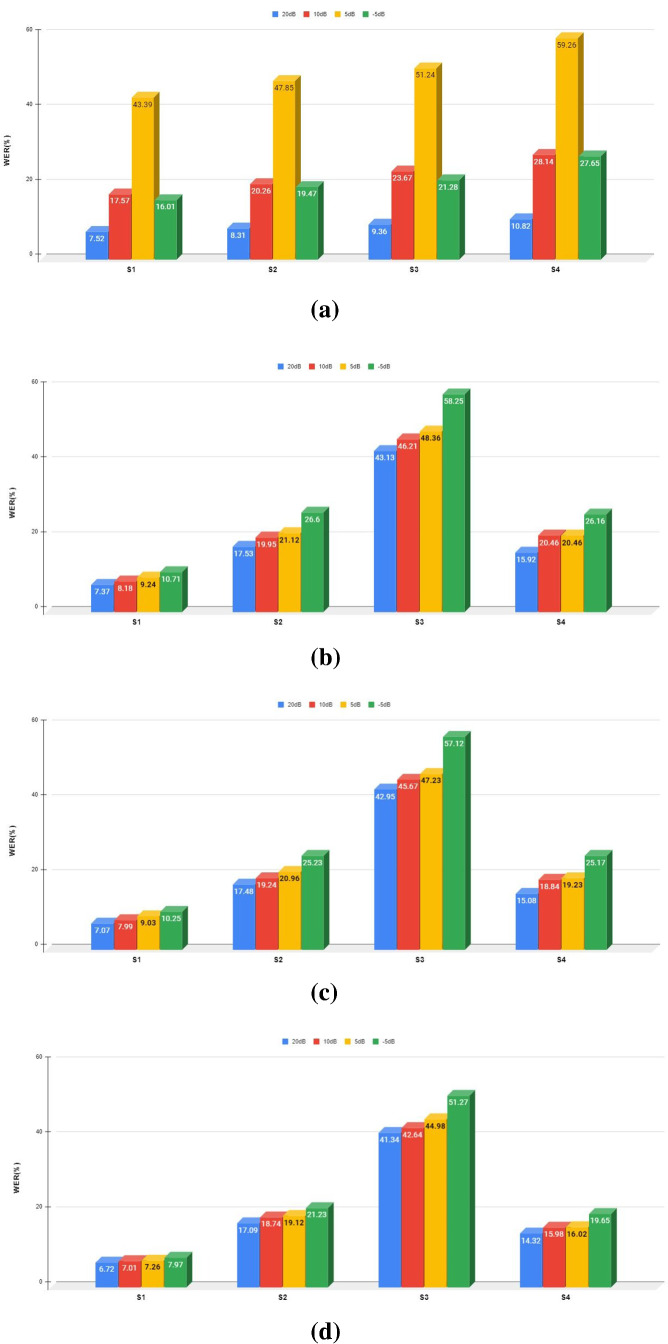
**a** WER obtained on utilization of MFCC feature extraction technique on both matched and mismatched systems. **b** WER obtained on utilization of RASTA-PLP feature extraction technique on both matched and mismatched systems. **c** WER obtained on utilization of GFCC feature extraction technique on both matched and mismatched systems. **d** WER obtained on utilization of PNCC feature extraction technique on both matched and mismatched systems

To better understand the impact of environment variation between clean trains and varying test conditions, we plot all system WER performance using four different front-end feature vector approaches. Figure 7a–d shows the system WER obtained after each noise level in dB tested on DNN–HMM classifier using four front-end feature extraction approaches of MFCC, GFCC PNCC and RASTA-PLP, respectively. First MFCC is evaluated which is found to be efficient in clean test signals but it is not robust to noisy test signals. Second, medium SNR-based noisy test signals are evaluated well with GFCC. At lower SNR’s both GFCC and RASTA-PLP performance are degraded so it is not worth in producing desired output. Apart, RASTA-PLP is found to be beneficial in only reduction of convolution noise at intermediate SNR values. Finally, PNCC performed asymmetric filtering which is found to be beneficial in suppressing background excitation and performing temporal masking. It can be noticed that PNCC performed well at large as well as less noisy dataset, whereas other approaches failed to achieve accuracy somehow at lower or upper SNRs.

It seems that in real application conditions where it is not mandatory to have a clean test signal, every signal may have a certain level of noise so finally PNCC worked well with large noisy dataset systems whereas MFCC with only clean test conditions. While there is a small benefit with GFCC has been also noticed with a limited SNR value within a middle range noisy or clean system with a smaller gain than noisy systems tested with MFCC.

### Performance evaluations on random noise-based training data augmentation

To further enhance the performance of the systems, each train system is augmented with synthetically induced noise signals. This pooling results in an enhanced train system which indulges the characteristics of different noisy signals at different SNR levels. The best signal accuracy obtained from Fig. 7a–d is taken into consideration that has been experimented on both clean test and noisy test conditions. The matched or mismatched systems accuracy has been enhanced by four front-end approaches. After training on augmented data in each four systems, it has been analyzed from Table 4 that S1–S4 has a R.I. of 36.41%, 28.94%, 25.01% and 29.45% using PNCC approach. In preliminary experiments, we experimented each test signal at different SNR’s only. But to directly measure the impact of each individual front-end approach both clean and noisy test speech is provided to every individual augmented train set. In a control mixing of noisy test sets, we found that PNCC outperformed in comparison to that of all other front-end approaches.

**Table 4:** WER obtained on noise augmented train set using varying front-end approaches

Training set	MFCC		RASTA-PLP		GFCC		PNCC	
	Clean test set	Noisy test set	Clean test set	Noisy test set	Clean test set	Noisy test set	Clean test set	Noisy test set
S1 + random noise	7.32	9.42	7.01	8.25	6.50	7.12	5.99	6.04
S2 + random noise	15.61	18.55	15.07	17.42	14.61	15.66	13.24	13.31
S3 + random noise	42.21	49.62	41.93	47.26	40.15	44.13	37.21	39.23
S4 + random noise	14.18	17.96	13.86	16.51	13.16	14.53	12.67	12.69

### Performance analysis of discriminative analysis under noisy and clean conditions when adult and adult–child in training set

The experiments thus far worked on the front-end system but to produce the better output system training and feature classification on the train dataset also plays an important role. We now evaluate the best output of Table 3 above by fixing PNCC as a front-end approach only. To further boost the system performance, initially, optimal value of MMI iteration has been performed. It is evaluated on both environment test sets using S1 and S4 systems only. The purpose of selecting these two systems is that in S1 only the adult test is evaluated, but in S2 and S3, only the child is evaluated which performs better in only the S4 system. Table 5 shows that the S1 and S4 systems performed better at iteration value of 3 in clean and 4 in noisy test sets. It obtained a RI of 6.01% and 4.26% in clean and a RI of 8.94% and 4.88% in noisy test sets in both the systems. In addition, we also conclude that parameter optimization using MMI performed better than conventional MLE approach which is employed with default DNN–HMM model. It contributed due to occupancy of probability in case of model-space training by reducing the impact of likelihood. It is only possible by maximizing the numerator and minimizing the denominator values in each lattice.

**Table 5:** WER obtained on varying no of MMI iterations in matched and mismatched systems using clean and noisy test sets

No. of iterations (MMI)	WER (%)			
	Clean test set		Noisy test set	
	S1	S4	S1	S4
1	6.97	14.25	6.89	13.89
2	6.25	13.12	5.97	12.27
3	**5.63**	12.65	**5.5**	12.14
4	5.68	**12.13**	5.61	**12.07**
5	5.59	12.19	5.48	12.12
6	5.58	12.17	5.51	12.09
7	5.59	12.18	5.51	12.11
8	5.59	12.17	5.5	12.11

Bold values imply a reduced word error rate (WER) that will be carried through

Similar to MMI further MPE is also tested which generally employs large segment feature information. It basically processes on small string values to further experiment phone level utilization. While performing such optimization, different LM based on 1, 2, 3 or 4-g are evaluated as in Table 6 on each test set of S1 and S4 systems. While performing such optimization, it can be analyzed that it generates improved results on 3-gm LM with a R.I. of 2.54% on S1 and 3.56% on S4.

**Table 6:** WER obtained on varying no of LM models with MPE training criteria in matched and mismatched systems using clean and noisy test sets

LM	WER (%)			
	Clean test set		Noisy test set	
	S1	S4	S1	S4
1-g	7.56	14.21	7.52	14.04
2-g	6.61	12.27	6.47	12.02
3-g	**5.57**	**11.74**	**5.39**	**11.64**
4-g	5.59	11.81	5.4	11.66

Bold values imply a reduced word error rate (WER) that will be carried through

To select an efficient optimization approach, further boost value is tried to be selected from different boost parameters of bMMI approach as in Eq. (17). Its impact has been studied and Table 7 depicts that an optimal boost value of 0.15 in S1 and S4 clean and 0.2 in S1 and S4 noisy test set has been evaluated to obtain an efficient boosting value of numerator lattice. It has been performed by boosting the word sequence likelihood.

**Table 7:** WER obtained on varying boost factor with MMI approach in matched and mismatched systems in clean and noisy test sets

Boost factor	WER (%)			
	Clean test set		Noisy test set	
	S1	S4	S1	S4
0 (mmi)	5.63	12.13	5.5	12.07
0.05	5.6	12.04	5.47	12.01
0.1	5.52	11.93	5.43	11.87
0.15	**5.49**	11.89	**5.39**	11.73
0.2	5.51	**11.74**	5.41	**11.64**
0.25	5.53	11.76	5.44	11.66

Bold values imply a reduced word error rate (WER) that will be carried through

In summary, it can be concluded that all the above parameter-tuned approaches are evaluated with DNN on each system (S1 and S2 on different test sets). To further enhance the system performance, sMBR is employed where each lattice is produced on each HMM state. It is possible by framing lattice on each corresponding state. It helps in evaluation of average path using MBR posterior probabilities. It is employed on denominator lattices by minimizing error rate in reference to different levels of granularity. Table 8 shows that each lattice-based parameter optimization approach has achieved a certain level of system performance improvement. These tuned optimization-based DNN acoustic models as in Table 8 achieved a RI of 10.58% in case of S1 and RI of 14.34% with DNN-sMBR model in comparison to other parameter-optimized approaches in each system. It helped in improved matched and mismatched systems with less training complexity.

**Table 8:** An overview of WER obtained discriminative training approaches in matched and mismatched systems using clean and noisy test sets

System type	WER (%)			
	Clean test set		Noisy test set	
	S1	S4	S1	S4
DNN-MMI	5.63	12.13	5.5	12.07
DNN-MPE	5.57	11.92	5.46	11.76
DNN-bMMI	5.49	11.74	5.39	11.64
DNN-sMBR	4.97	10.17	4.82	9.97

### Performance analysis of gender-based selection under mismatched system on clean and noisy test dataset

The experiments thus far worked on the mismatched conditions where enough present adult data are mixed with low-resource children dataset to resolve the problem of data scarcity. In these set of experiments employing discriminative training techniques, the adequate measure for the gender-based selection is further experimented. These set of experiments help in finding the adequate gender-selection considering female adult and male dataset individually for testing the familiarization and likelihood with children dataset. Therefore, from the Table 9, it can be observed that the female adult data have adapted more with the children dataset such that a certain level of system performance improvement in contrast to adult male dataset under mismatched conditions has been obtained. The reason for such improved performance is much familiarized characteristics of children and female including the vocal tract length differences, speaking rates and pitch concerning the same. The female-based selection as in Table 9 achieved a RI of 1.18% and 1.02% with DNN-sMBR model in contrast to S4 system being evaluated in Table 8 under clean and noisy test conditions.

**Table 9:** An overview of WER obtained of discriminative training approaches employing gender-based selection on mismatched system using clean and noisy test sets

System type	WER (%)			
	Clean test set		Noisy test set	
	Female adult + child	Male adult + child	Female adult + child	Male adult + child
DNN-MMI	11.81	12.34	11.69	12.26
DNN-MPE	11.85	12.32	11.65	11.82
DNN-bMMI	11.57	11.80	11.44	11.85
DNN-sMBR	10.05	11.01	9.85	10.34

### Performance analysis under augmentation adult and adult–child in training set

To avoid the issue of data scarcity and inter-speaker variations that are caused due to less child train dataset and variations caused due to vocal tract length of adult and child. We first artificially increased training dataset by pooling of original S1 and S2 speech through mixing of artificial noise along with three-way perturbation that make three-time training data which tried to make full utilization of DNN-sMBR and TDNN-sMBR approach. TDNN-sMBR-based discriminative acoustic training has outperformed DNN-sMBR as per the evaluations being detailed in Table 10. Likewise, the system has been evaluated on the PNCC front-end approach using a different test set. The system also tested with and without vocal tract length normalization approach. This normalization tried to overcome the issue of mismatched training and test speech signals. It can be performed by normalizing only the test set by processing it on without normalizing the train set. Therefore, TDNN-sMBR modeling results in an overall RI of 40.18%, 47.51%, and 47.64% on S1 ASR system, S4 ASR system and female adult selected ASR system, respectively.

**Table 10:** An overview of WER obtained from perturbation training using PNCC and VTLN approaches in matched and mismatched systems using clean and noisy test sets

Training set	Classifier type	PNCC		PNCC + VTLN	
		Clean test set	Noisy test set	Clean test set	Noisy test set
S1 + noise + 3-way	DNN	4.64	4.68	4.37	4.48
S4 + noise + 3-way		9.38	9.24	8.82	8.64
Female adult + noise + 3-way		9.31	9.18	8.71	8.62
S1 + noise + 3-way	TDNN	4.18	4.27	3.90	4.02
S4 + noise + 3-way		8.89	8.65	8.26	8.10
Female adult + noise + 3-way		8.85	8.59	8.20	8.08

### Performance analysis based on gender-based spectral augmentation under mismatched conditions

**Fig. 8:**
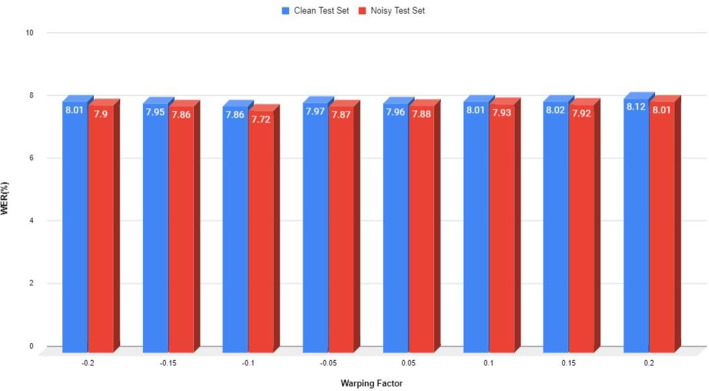
WER obtained on utilization of spectral warped adult female dataset employed with PNCC + VTLN-based feature extraction technique on mismatched systems

The use of the normalization methodology aided in the optimization of signal frequency axes via an appropriately chosen warping factor. Aberrations induced by changes in voice tract length, on the other hand, can be represented by a simple linear warping within the spectral domain of audio signals. Thus, the methodology of spectral-based augmentation has been applied in the context of speaker-independent ASR, wherein speaker-independent HMMs are developed using syllables from a gender-based selection of adult speakers. The spectral augmentation approach applied on gender dataset using PNCC + VTLN- on TDNN-based classifier has enhanced the system’s performance, as shown in Fig. 8. The optimum findings are obtained at − 0.1, − 0.05, and + 0.05 values of the warping factor. It has been reported that an adequate development of speaker-independent HMM system with sufficient gender selection is produced over a frequency-adjusted feature. Furthermore, the experimentation tried to consider the PNCC + VTLN feature set using TDNN classifier on permutation mixture of optimum spectral warping factors. Table 11 details the combinational values of spectral warping factor, i.e., − 0.1 and 0.05 which resulted into an enhanced performance through RI of 5.49% and 12.62% in both clean and noisy conditions. Thus, the transformation matrix variant on gender selection can be thought of as a bank of FIR filters that can be effectively utilized such that the impulse responses while adapting from adult to children are easily available given that the spectral warping transform is not time invariant.

**Table 11:** An overview of WER obtained after combining spectral warping technique through mismatched systems on clean and noisy test sets

Training set		Perturbation type	Classifier type	PNCC + VTLN	
Noise augmented dataset	Warping factor			Clean test set	Noisy test set
Female adult + noise	–	Three-way	TDNN	8.20	8.08
	− 0.1 + 0.05			7.75	7.06
	− 0.1 ± 0.05			7.78	7.14
	0.05 ± 0.05			7.86	7.34

### Comparative performance analysis of proposed system architecture with earlier implemented approaches

Automatically recognizing speech in children’s speech under certain inconsistencies including mismatched conditions, i.e., on adult speech models, is a well-known difficulty, considering the variations in language of adults and children. The study on children’s speech is almost zero concerning low-resource languages as the children’s speech and labeling details are unavailable. Likewise, the developed ASR systems are normally trained on sufficiently accessible or self-created spoken information for adults, and are checked on child speech data to solve the above problems. In addition, the geometry of vocal organs in both children and adults (smaller in children) differs significantly, resulting in the scaling of the fundamental frequency or pitch. The accuracy of stochastic determination generally depends on the assumption of mathematical models matching a signal input. However, computation limitations on a handy amount of data necessary to accurately adjust pattern parameters in sequence training are modest in the case of child speech recognition. The problem of data scarcity is evaluated by training the ASR system on suitably useable or automatically generated adult language data and child speech data training. Therefore, Table 12 summarizes the already implemented approaches concerning children and low-resource languages in contrast to the proposed system architecture employing PNCC + VTLN feature extraction on TDNN-sMBR architecture.

**Table 12:** Comparative analysis and summarization of earlier implemented approaches in constant to proposed system architecture

Author details	Dataset details	Methodologies	Summary
Kadyan et al. [13]	Punjabi adult corpora constituting continuous and phonetically rich sentences	MFCC; GFCC-based hybrid DNN–HMM and GMM–HMM modeling	The reduction in size, vector knowledge de-correlation and speaker heterogeneity are being discussed by the researcher employing LDA, transition probability, speaker adaptive tri-phones, highest probability, linear regression adaptation models. In two hybrid classifiers, the accuracy of the interconnected and ongoing Punjabi voice corpus is studied. GMM–HMM and DNN–HMM with the experimental configuration detailing significant RI of 4–5% and 1–3%, respectively
Shivakumar et al. [5]	English language children dataset employing transfer learning	MFCC-based GMM–HMM and DNN–HMM-based modeling	The paper presents a systematic and an extensive analysis of the proposed transfer learning technique considering the key factors affecting children’s speech recognition from prior literature. Evaluations are presented by making the comparisons of earlier GMM–HMM and the newer DNN Models such that the author had experimented for the detailed effectiveness of standard adaptation techniques versus transfer learning
Kumar et al. [42]	Adult data comprising of 13,218 Punjabi words with over 200 min of recorded speech	MFCC feature extraction technique	In this paper, the author has experimented for auto-denoising method employing the novel Corpus Optimization Algorithm on the Punjabi language corpus. At the same time, for 13,218 Punjabi words, the WER was lowered to 5.8%. Likewise, some other important factors such as the total probability per frame and the convergence ratio spanning different iterations for obtainable Gaussian mixtures has also been evaluated and consequently the improved performance of the system has been relatively being suggested
Gretter et al. [43]	TLT-school corpora containing Italian children recorded English dataset	Metrics for collection of adequate children data based upon good pronunciation vs bad pronunciation	The researchers have maintained for the collection of corpuses corresponding to students between 9 and 16 years of age, students from elementary, secondary and secondary schools, was registered in 2017 and 2018. Both statements have been obtained by human experts with regard to certain predefined ability measures
Kadyan et al. [44]	Punjabi children speech corpora	MFCC; MFFC + Pitch; MFCC + Pitch + VTLN-based DNN–HMM modeling	Substantially lower error rates from an increase in off-domain data dependent on prosody modifications has been experimented by the researcher. Furthermore, the authors analyzed the impact of changing the number of senones, the number of hidden nodes and layers, and the early stagnation, which resulted in a relative improvement of 32.1% (RI) in contrast to the baseline structure of different senones
Dua et al. [45]	Hindi speech corpora	Discriminative training based on MPE through variations among the quantity of Gaussian mixtures	The researcher has trained speech recognition through interpolation of language model and discriminative approaches. They achieved a relative improvement of 85.45 under clean and 82.95 under noisy conditions
Kadyan et al. [46]	Punjabi adult corpora comprising of isolated and phonetically rich sentences	MFCC coupled bottleneck features based on Tandem-NN acoustic modeling	In this paper, the authors have processed context-independent input speech signal information through utilization of bottleneck characteristics. Further noisy data have been handled and experimental results revealed that under clean and noisy settings a Tandem-NN system achieved a RI of 13.53% as compared to the Baseline system
Dua et al. [47]	Hindi continuous sentences speech corpora and noise augmented dataset	Use of noise-resistant integrated features and an improved HMM model for the development of discriminatively trained speech recognition system	The suggested study has examined that with MF-PLP and MF-GFCC alone or integrated feature vectors results into large performance improvement
Kumar and Aggarwal [48]	Two low-resource Indo-Aryan family languages including Hindi and Marathi	Integrated features vector with RNN being employed on Hindi ASR system utilizing MLLR and constrained-MLLR)	The researcher experimented 256 Gaussian mixtures corresponding to every HMM state using discriminatively trained method of MMI and MPE. The experiments showcased that the discriminative training has been improved in comparison to baseline system by 3%
Bawa et al. [1]	Gender-based selection under mismatched conditions	MFCC; GFCC-based DNN–HMM modeling	The study attempts to create Punjabi Children ASR in mismatched parameters via noise-robust techniques such as the MFCC or GFCC. Accordingly, acoustic and phonetic differences between adults and children are managed by gender-based selection of adult data and subsequent acoustic variability across speakers in training and test conditions are normalized by means of the VTLN with 30.94% of RI in comparison to the baseline system
Proposed approach	Punjabi adult and children under mismatched conditions	PNCC; PNCC + VTLN-based DNN-sMBR and TDNN-sMBR modeling; gender-based selection; spectral augmentation	(i) The results demonstrate that ASR frames examined on PNCC + VTLN techniques are only successful when testing it on sMBR optimized acoustic models. The outcomes of these experiments shown that an overall RI of 40.18%, 47.51%, and 47.64% are achieved, respectively, with S1 and S4 ASR systems and female adult-selected ASR system (ii) Second, the gender-based spectral augmentation has led to an enhanced performance improvement of 49.87% in comparison to the baseline system

## Conclusion

In this study, heterogeneous front-end: MFCC-, GFCC-, RASTA-PLP-, and PNCC-based robust ASR framework has been systematically presented that provides better accuracy using various parameter optimized sequence-discriminative training approaches on acoustic modeling phase. These approaches have been implemented on large adult speech and very low child speech on true matched and mismatched systems. Further, the issue of data scarcity caused due to small original train speech is resolved using out-domain augmentation strategy. These results in large training complexity because of the multi-style data augmentation strategy employed through pooling of original speech and noise injected at different SNR level synthetic speech. It resulted into over fitting and confusion of acoustic model information, so it is additionally processed using parameter optimization of feature vectors by MMI, MPE, bMMI, and sMBR approaches which are processed on the basis of lattice generation, and adjustments of learning rates. It tried to be demonstrated for developing an effective training system. Moreover, the adequate gender-based selection concerning adult data has solved for the problem of the data shortage as well as reduced differences of acoustic mismatched parameters including frequency and vocal tract length has led to substantial improved performance of the system. Further, this paper also included additional inter-speaker variability reduction methods between adult and child speech using the VTLN approach in the test set only. It is found to be efficient in normalization of training and testing dataset differences caused due to varying vocal length through optimal selection of warp factor. The experiment results showed that ASR frameworks investigated on PNCC + VTLN approaches are found to be effective with only test normalized systems on TDNN-sMBR-optimized acoustic models. However, the results show a relative improvement of 47.51% on mismatched, 40.18% on matched systems and 49.87% on adequate gender-selected systems than other ASR frameworks, respectively. Further work can be extended by speech rate rhythmically parameter-based classification approach for normalization of individual adult and child speech trained systems on true matched and semi- or mismatched conditions on the basis of test speech. Further to that, a robust switch to process separate clean and noisy environment dataset is also required to implement an efficient front-end approach that wishes to address the drawbacks of the proposed approach.
